# Supramolecularly engineered bacteria mediated calcium overload and immunotherapy of tumors

**DOI:** 10.7150/thno.99931

**Published:** 2024-10-07

**Authors:** Beibei Xie, Linmiao Dong, Leo Wang, Ruibing Wang, Chunlai Li

**Affiliations:** 1 Department of Liver Surgery, Renji Hospital, Shanghai Jiao Tong University School of Medicine; Shanghai Engineering Research Center of Transplantation and Immunology, Shanghai 200127, China.; 2 State Key Laboratory of Quality Research in Chinese Medicine, Institute of Chinese Medical Sciences, University of Macau, Taipa, Macau 999078, China.; 3 Kitsilano Secondary School, Vancouver, BC V6K 2J6, Canada.

**Keywords:** supramolecular assembly, engineered bacteria, calcium-overload, immunotherapy

## Abstract

Intracellular Ca^2+^ nanogenerators, such as calcium carbonate, calcium peroxide, and calcium phosphate nanoparticles, have shown promise in calcium overload-mediated tumor therapy. However, their effectiveness is often hampered by poor targeting, low accumulation, and limited penetration into tumor cells, leading to suboptimal therapeutic outcomes. This strategy aims to achieve synergistic Ca^2+^ overload and immunotherapy of tumors.

**Methods:** A supramolecular conjugate of engineered living bacteria (facultative anaerobic Salmonella typhimurium VNP20009, VNP) with CaCO_3_ nanoparticles was developed for targeted delivery of curcumin-loaded CaCO_3_ into tumors.

**Results:** Both CaCO_3_ nanoparticles and the loaded Ca^2+^ efflux inhibiting agent, curcumin (CUR), demonstrated significant enhancement of intracellular Ca^2+^ overload, resulting in apoptosis of tumor cells via mitochondrial dysfunction. Moreover, VNP exhibited excellent tumor-targeting ability, colonization in tumor tissues, and anticancer activity with minimal side effects.

**Conclusion:** The conjugate of VNP and CaCO_3_ not only enhances the efficiency of common cancer treatments but also synergizes Ca^2+^ overload with cancer immunotherapy, thereby offering a promising approach for improving therapeutic outcomes in cancer treatment.

## Introduction

Cancer remains one of the leading causes of mortality worldwide, posing a significant threat to human health 1, 2. Despite the widespread use of traditional approaches like surgery, radiotherapy, and chemotherapy in cancer treatment, their associated side effects including physical trauma, immune compromise, and drug resistance greatly diminish therapeutic efficacy 3, 4. Consequently, there is an urgent imperative to explore alternative strategies for tumor treatment that offer both high efficiency and minimal adverse effects.

Ca^2+^ overload has been shown to induce mitochondrial disorders and disrupt mitochondrial respiration 5, 6, ultimately leading to cancer cell apoptosis 7-9. Recently, intracellular Ca^2+^ nanogenerators such as calcium carbonate (CaCO_3_) 5, 10, calcium peroxide 11, and calcium phosphate nanoparticles have emerged as promising agents for tumor therapy by inducing Ca^2+^ overload-mediated cell apoptosis 12. However, challenges such as poor targeting, low accumulation, and inadequate Ca^2+^ overload in tumor cells severely constrain the therapeutic efficacy of these intracellular Ca^2+^ nanogenerators.

The anaerobic, eutrophic, and immunosuppressive microenvironment of tumors provides an ideal habitat for bacterial localization 13-18. Bacteria have been extensively utilized as effective drug delivery vehicles, demonstrating remarkable targeting ability and minimal side effects 19-22. Moreover, colonization of bacteria within tumors can trigger both innate and adaptive immune responses, leading to the nonspecific elimination of heterogeneous tumor cells 23, 24. Consequently, leveraging bacteria as delivery vehicles significantly enhances the accumulation and penetration of therapeutic agents within tumors, thereby improving the overall efficacy of tumor therapy.

Herein, we developed a supramolecular conjugate of living bacteria and Ca^2+^ nanogenerators through a host-guest interaction for the synergistic Ca^2+^ overload and immunotherapy against tumors with improved specificity and efficacy. Living facultative anaerobic *Salmonella typhimurium* VNP20009 (VNP) with well tumor-targeting ability, anticancer activity and safety were selected as a delivery vehicle 14, 25, 26. Polydopamine (PDA) modified CaCO_3_ nanoparticles (CaP) loaded with curcumin (CUR), abbreated as CaPC, was prepared as a intracellular Ca^2+^ nanogenerator. A supramolecular guest precursor (adamantine, ADA) was covalently conjugated with CaPC (CaPCA) through esterification. As a supramolecular host precursor, cucurbit[7]uril (CB^7^) was conjugated onto the VNP surface via a simple lipid ligand membrane-insertion to obtain the CB^7^ modified VNP (VNP-CB^7^). The supramolecular conjugate of CaPCA and VNP-CB^7^, abbreviated as CaPCAV, was obtained through strong host-guest interaction between CB^7^ and ADA (Ka >10^6^·M^-1^). CaPCAV could specifically accumulate at tumor sites through the excellent tumor-tropism of VNP. The slightly acidic environments of tumor cells induced the CaCO_3_ nanoparticles to release a mass of Ca^2+^, which could induce the damage of mitochondria (white color), resulting in intramitochondrial Ca^2+^ overload. Meanwhile, the released CUR, as a Ca^2+^ efflux inhibiting agent, could further promote the release of Ca^2+^ from the endoplasmic reticulum to the cytoplasm and hence inhibited Ca^2+^ efflux 12, 27.The colonized VNP with well tumor-targeting ability, anticancer activity and safety, in tumor sites could promote M1 polarization and induce the adaptive immune responses 28, improving immunotherapeutic efficacy of tumors. Therefore, the conjugate of VNP and CaCO_3_ could achieve the effective therapy of tumors with minimal side effects through synergizing the enhanced Ca^2+^ overload and cancer immunotherapy.

## Results and Discussion

### Preparation and characterization of CaPC and CaPCA

**Figure 2:**
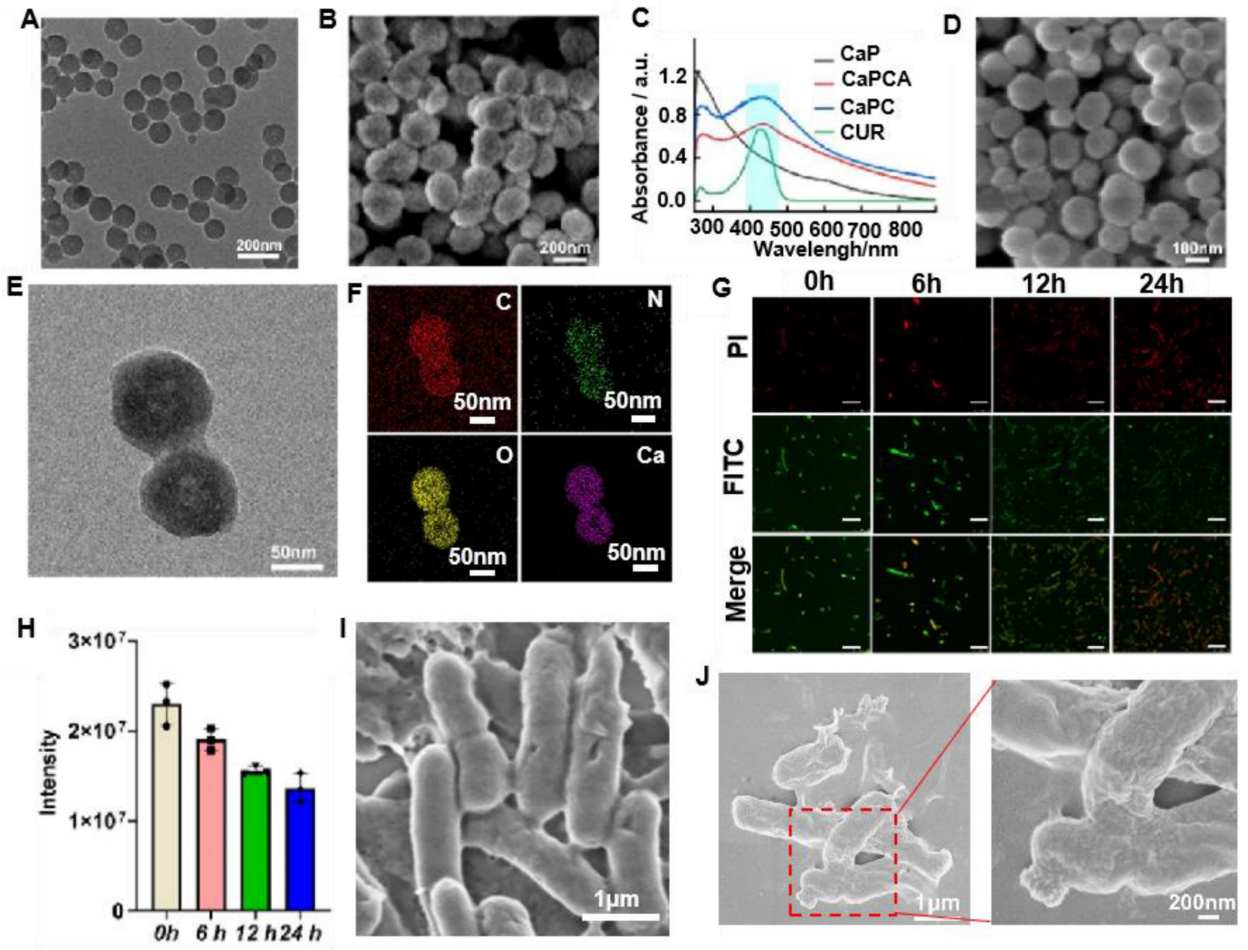
Preparation and characterization of CaPC, CaPCA, VNP-CB^7^, CaPCAV. A) TEM and B) SEM images of CaPC. C) UV-Vis absorption spectra of CUR, CaP, CaPC and CaPCA. D) SEM, E) TEM image and F) corresponding elemental mapping images of C, N, O, Ca element of CaPCA. G) VNP-CB^7^ were modified with FITC-ADA and observed by CLSM at 0, 6, 12 and 24 h, as well as H) the corresponding fluorescence intensity of FITC. The scale bar was 5 μm. The SEM image of I) VNP and J) CaPCAV.

CaPC was prepared through a reported method 29. Briefly, ammonia (NH_3_) and carbon dioxide (CO_2_) released from the decomposition of ammonium bicarbonate (NH_4_HCO_3_) in a sealed container, were continuously diffused into the mixture of calcium chloride (CaCl_2_), dopamine (DA) and CUR to create an alkaline environment and served as a source of carbonate ions (CO_3_^2-^), respectively. Then, the simultaneous polymerization of DA to PDA and the formation of CaCO_3_ resulted in the formation of CaPC. Both TEM (Figure 2A) and SEM images (Figure 2B) indicated that CaPC exhibited a sphere morphology. The results of the TEM (Figure 2B) and dynamic laser scattering (Figure S1) indicated that the diameter of CaPC was about 140 nm. The successful loading of CUR in CaPC was confirmed by the appearance of the CUR absorption peak at 427 nm (Figure 2C).

The supramolecular precursor, ADA was covalently conjugated with CaPC through esterification between ADA modified with carboxyl (COOH-ADA) with PDA to obtain the CaPCA (Figure S2). The successful conjugation of ADA on CaPC was confirmed by ^1^H NMR (Figure S3). The decreased Zeta potential also confirmed the conjugation of ADA with CaPC (Figure S4). After the conjugation of ADA, the CaPCA still exhibited the same sphere morphology and size with CaPC through the observation of SEM (Figure 2D) and TEM images (Figure 2E). The uniform distribution of C, N, O and Ca element in the elemental mapping spectra of CaPCA further confirmed the coating of PDA and ADA on CaCO_3_ (Figure 2F). After 7 days, the very modest size variation of CaPCA confirmed the good hydrodynamic stability of CaPCA (Figure S5). The stability of particles in fetal bovine serum (FBS) is an important parameter to assess the stealth property of nanoparticles. The negligible size variation of CaPCA after incubation with FBS for 12 h indicated the immune compatibility and stealth property of CaPCA (Figure S6).

### Preparation and characterization of VNP-CB^7^

CB^7^ modified VNP was obtained through our previously reported work (Figure S7) 30. Firstly, CB^7^ was conjugated with a lipid molecule,1,2-distearoyl-sn-glycero-3-phosphoethanolamine-poly(ethyleneglycol) (DSPE-PEG) 31-34, through a thiolene click reaction (Figure S5). The successful conjugation of CB^7^ with DSPE-PEG (DSPE-PEG-CB^7^) was confirmed by the ^1^H NMR spectra (Figure S8). Subsequently, VNP-CB^7^ was obtained through the co-incubation of VNP with DSPE-PEG-CB^7^. Finally, the ferrocene was incubated with VNP-CB^7^ through the strong host-guest interactions between CB^7^ and ferrocene to measure the concentration of CB^7^ on VNP. The concentration of CB^7^ on VNP was about 4 nmol of CB^7^ per 10^7^ CFU of VNP via calculating iron content. ADA modified with fluorescein isothiocyanate (FITC-ADA) was used for labeling CB^7^ on VNP to evaluate the stability of membrane decoration of CB^7^ in VNP. After incubation for 6 h, a bright green fluorescence of FITC was observed and still maintained a high intensity of fluorescence for 24 h post-incubation (Figure 2G and 2H), which indicated the high stability of membrane decoration of CB^7^ on VNP.

### Preparation and characterization of CaPCAV

**Figure 3:**
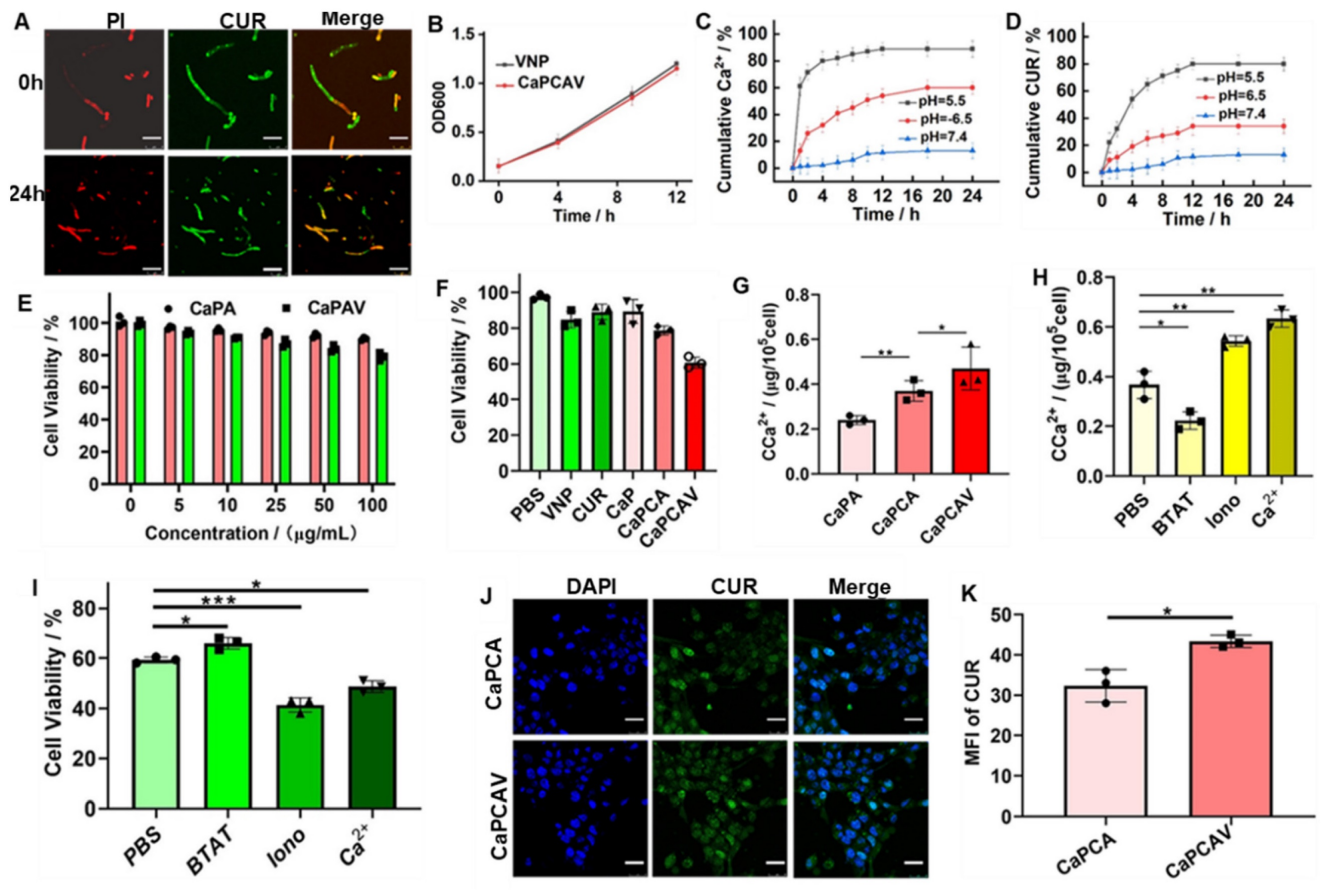
The cytotoxicity and mechanism of Ca^2+^ overload induced by CaPCAV *in vitro*. A) CLSM images of CaPCAV after incubation for 0 and 24 h, the scar bar was 5 μm. B) The proliferation profiles of VNP and CaPCAV for up to 12 h. Release profiles of C) Ca^2+^ and D) CUR from CaPCAV in PBS solutions with different pH. E) The viability of LO2 cells treated with CaPA and CaPAV containing the different concentrations of CaP. F) The viability of 4T1 cells after 24 h incubation with various formulations containing equivalent concentration of CUR (20 μgꞏmL^-1^), CaP (100 μgꞏmL^-1^) and VNP (10^6^ CFU). G) The content of intracellular Ca^2+^ of 4T1 cells incubated with CaPA, CaPCA, CaPCAV containing the equivalent amout of CaP (100 μgꞏmL^-1^) and CUR (20 μgꞏmL^-1^) after 24 h, respectively. H) The content of intracellular Ca^2+^ in 4T1 cells treated with CaPCAV+PBS, CaPCAV+Iono, and CaPCAV+ BATM, respectively, after 24 h. I) The viability of 4T1 cells after 24 h incubated with CaPCAV+PBS, CaPCAV+Iono, and CaPCAV+ BATM, respectively. J) CLSM images of 4T1 cells after 12 h cellular internalization of CaPCA, CaPCVA. the scar bar was 5 μm. K) Quantitative analysis of the fluorescence intensities of CUR. Data are shown as mean ± standard deviation (SD) (n = 3; *P < 0.05, **P < 0.01, ***P < 0.001).

CaPCAV was obtained through strong host-guest (CB^7^-ADA) interaction between CaPCA and VNP-CB^7^. VNP exhibited the smooth surface (Figure 2I), while CaPCAV exhibited the rough surface due to decoration of CaCO_3_ on VNP (Figure 2J). The loading amount of CaCO_3_ nanoparticles was calculated to be about 1.5 mg per 10^5^ CFU of VNP through weight difference method. For studying the stability of CaPCA on CaPCAV, PI with red fluorescence was used to mark VNP-CB^7^. After incubation for 24 h, CUR with green fluorescence still maintained a high intensity of fluorescence (Figure 3A), indicating the well stability of CaPCAV, which was beneficial for *in vivo* delivery. CaPCAV exhibited the similar proliferation profiles with VNP after incubation for 12 h (Figure 3B), indicating the negligible effect of CaPCA on the proliferation of VNP. The negligible size variation of CaPCAV after 7 days' incubation with PBS further confirmed the good hydrodynamic stability of CaPCAV (Figure S9).

### The acid-responsive release of Ca^2+^ and CUR

The loading ratio of CUR in CaPC was as high as 25.5%. After conjugation of ADA, the loading ratio of CUR in CaPCA was 24%, indicating the high stability of CUR in CaPCA during the process of esterification. CaPCA were immersed in different PBS solution to evaluate their behaviors of acid-responsive decomposition. CaPCA was stable in PBS at pH 7.4 (Figure S7A), slightly decomposed at pH 6.5 (Figure S7B), while almost completely decomposed at pH 5.5 (Figure S7C). CaPCAV also exhibited acid responsive release of Ca^2+^ (Figure 3C) and CUR (Figure 3D). The minor release of Ca^2+^ and CUR from CaPCAV was observed at neutral environment even after 24 h, while about 89% of Ca^2+^ and 80% of CUR were released from CaPCAV after 4 h under pH 5.5, respectively. The burst release of Ca^2+^ and CUR at acidic environment from CaPCAV was beneficial to Ca^2+^ overload inside tumor cells.

### *In vitro* cytotoxicity induced by Ca^2+^ overload and VNP

The carrier of CaPA and CaPAV had negligible cytotoxicity against LO2 cells even at the high concentrations of 100 μgꞏmL^-1^ (Figure 3E), indicating the well biocompatibility of CaPA and CaPAV. VNP with anti-cancer activity could prohibit the cell viability of 4T1 cells (Figure 3F) 14. CaPC and CaPCA exhibited stronger cytotoxicity against 4T1 cells than that of free CUR and CaP (Figure 3F), due to that the CaP and loaded CUR could synergistically enhance Ca^2+^ overload mediated apoptosis of tumor cells. CaPCA exhibited similar cytotoxicity against 4T1 cells with that of CaPC, indicating the coating of ADA has no effect on the cytotoxicity of CaPC against 4T1 cells. As expected, combing the cytotoxicity of VNP and Ca^2+^ overload mediated apoptosis induced by CaPCA, CaPCAV exhibited the highest cytotoxicity against 4T1 cells among all the studied groups.

The intracellular Ca^2+^ level was investigated via a Ca^2+^ quantitative assay kit to assess intracellular Ca^2+^ overload.^29^ Compared with CaPA, both CaPCA and CaPCAV exhibited a higher intracellular Ca^2+^ content (Figure 3G), due to the Ca^2+^ efflux inhibiting agent of CUR 35.The higher Ca^2+^ content in 4T1 cells treated with CaPCAV than that of CaPCA indicated that VNP could reduce the reflux of Ca^2+^, due to the reduced cell viability by VNP with anti-cancer activity. As a cell-permeant Ca^2+^ chelator, BAPTA-AM (BATM) could bind the intracellular Ca^2+^ and hence decrease the level of intracellular free Ca^2+^. Ionomycin (Iono) is an ionophore, which could specifically delivery extracellular Ca^2+^ into cells, resulting in the increase of intracellular Ca^2+^ 11. For investigating the effect of Ca^2+^ overload on cells viability, 4T1 cells were incubated with the CaPCVA containing PBS, BATM, Iono and CaCl_2_, respectively. The intracellular Ca^2+^ content treated with BAPTA-AM exhibited a dramatic decrease than that of PBS group (Figure 3H), which induced the corresponding increase of cell viability (Figure 3I). The intracellular Ca^2+^ content treated with Ca^2+^ and Iono exhibited an obvious increase than that of PBS group (Figure 3H), which induced the corresponding decrease of cell viability (Figure 3I). Compared with high intensity of green fluorescence of CUR in 4T1 cells treated with CaPCA (Figure 3J), 4T1 cells treated with CaPCAV exhibited the slightly higher fluorescence intensity of CUR (Figure 3K), which might be the reduced cell viability by VNP.

### The mitochondria damage induced by CaPCAV

**Figure 4:**
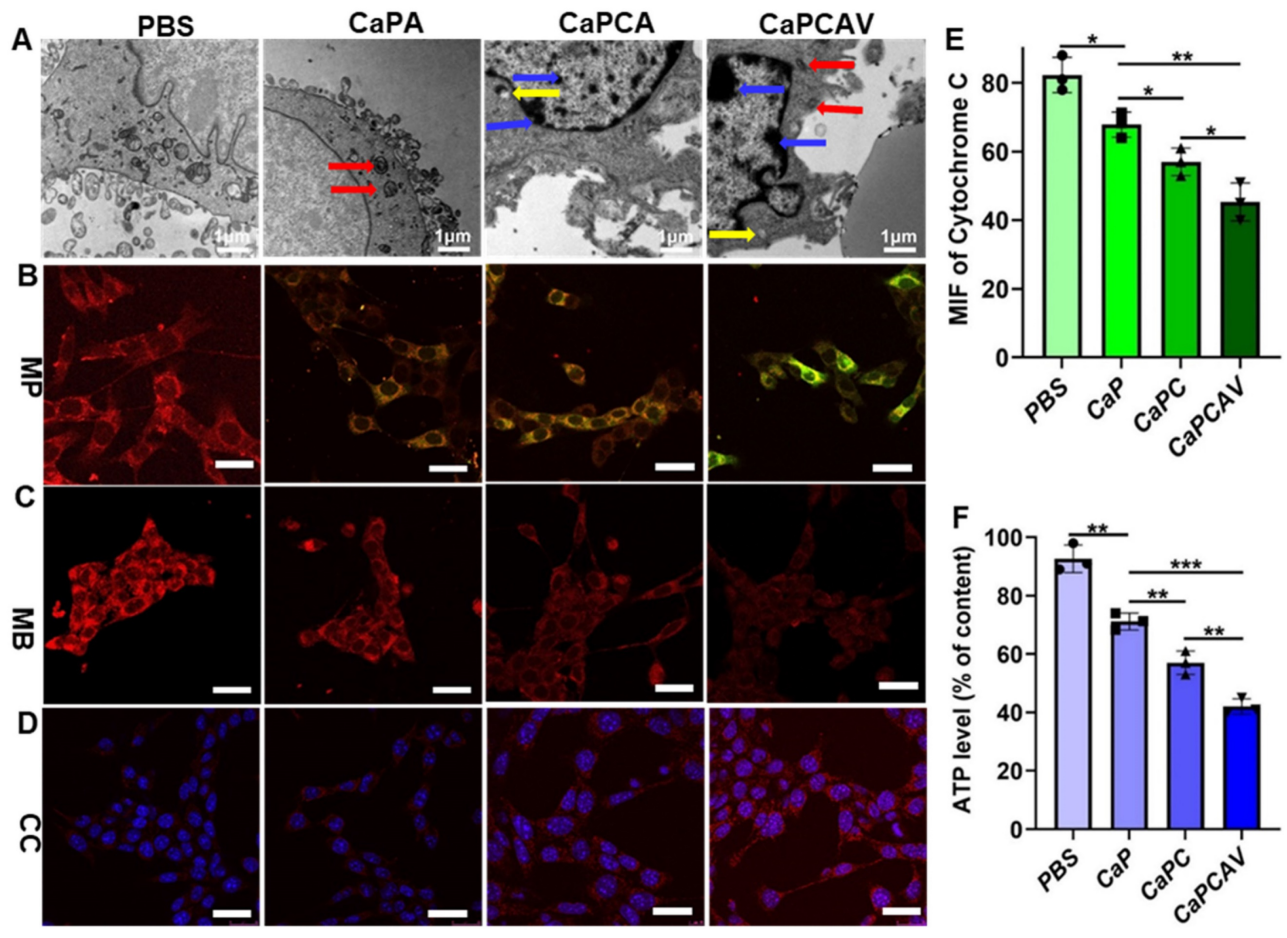
The effect of CaPCAV on mitochondria. A) Bio-TEM images, B) MMP, C) mitochondrial distributions and D) cytochrome C of 4T1 cells after incubation with PBS, CaPA, CaPCA and CaPCAV, respectively, for 12 h. The scale bar was 5 μm. E) The expression of cytochrome C and F) relative ATP levels of 4T1 cells after incubation with PBS, CaPA, CaPCA and CaPCAV, respectively, for 12 h. Data are presented as mean ± standard deviation (SD) (n = 3; *P < 0.05, **P < 0.01, ***P < 0.001).

The cell morphology was observed via bio-TEM to investigate the effect of Ca^2+^ overload on cells.^36^ Compared with the normal 4T1 cells, the CaPA, CaPCA and CaPCAV could induce distinct changes of cell morphology (Figure 4A). 4T1 cells treated with CaPCA exhibited more pronouced mitochondria damage than that of CaPA due to the enhanced Ca^2+^ overload induced by CUR, including the chromatin marginalization and densification into dense masses (marked by yellow arrow), the vesicular-like expansion (marked by blue arrows) and swollen mitochondria (marked by red arrows). 4T1 cells treated with CaPCAV showed the most serious mitochondria damage among all the groups, including more dense chromatin and vesicular-like mitochondria, which indicated that the conjugation of VNP could enhance mitochondria damage though intracellular Ca^2+^ overload.

Mitochondrial membrane potentials (MMP) and mitochondrial distributions were also investigated to further evaluate the mitochondria damage 37. MMP were assessed through staining with JC-1. JC-1 is a monomer with green fluorescence at low MMP but forming J-aggregates with red fluorescence at high MMP 29, 38. 4T1 cells treated with CaPA, CaPCA and CaPCAV exhibited lower MMP than that of PBS group (Figure 4B). Notably, 4T cells treated with CaPCAV exhibited the most obvious green fluorescence among all the groups, indicating the lowest MMP and most serious mitochondrial damage.

The mitochondrial distribution was assessed through staining with MitoTracker Deep Red FM 39. As shown in Figure 4C, 4T1 cells treated with different types of CaP exhibited weaker red fluorescence intensity than that of PBS group. The lowest red fluorescence was observed in 4T1 cells treated with CaPCAV, indicating the lowest number of mitochondria and the most serious mitochondrial damage. Cytochrome C could cleave pro-caspase-3 to form activated caspase-3, resulting in apoptosis of cells 40. Hence, the expression of cytochrome C were investigated through immunofluorescence staining to evaluate the cell apoptosis induced by mitochondrial damage (Figure 4D). 4T1 cells treated with CaPCAV exhibited the largest upregulation of cytochrome C among all the groups (Figure 4E), suggesting the cell apoptosis induced by mitochondrial damage.

The mitochondria damage could directly induce the decrease of intracellular adenosine triphosphate (ATP) levels 41. Therefore, intracellular ATP level in 4T1 cells was assessed through an ATP probe to further evaluate the damage of mitochondria. 4T1 cells treated with CaPCAV had the lowest ATP level among all the groups (Figure 4F), indicating the most severe mitochondrial damage.

### *In vitro* immune activation induced by CaPCAV

**Figure 5:**
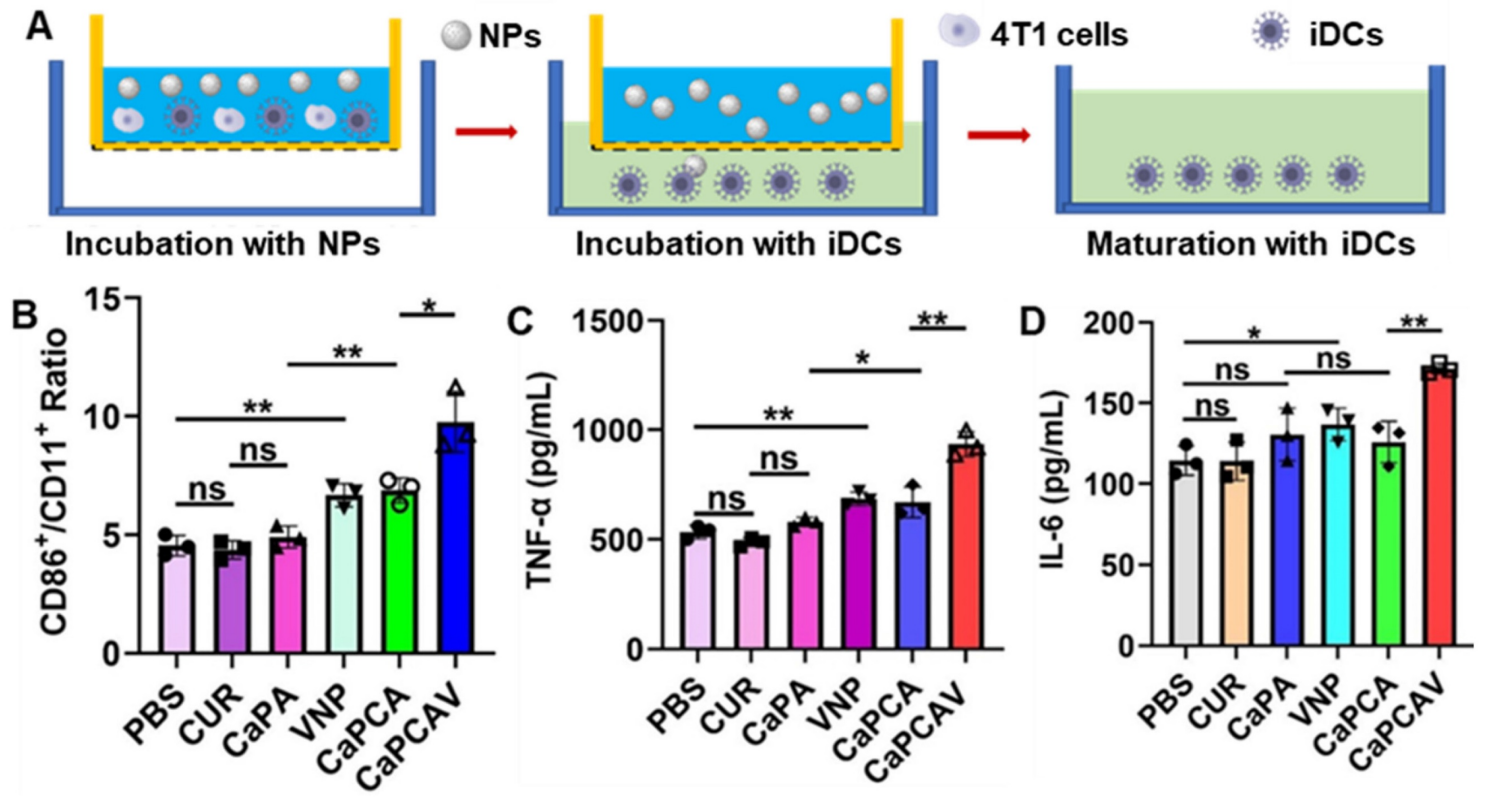
*In vitro* immune activation. A) The schematic co-culture system of 4T1 cells and bone marrow-derived iDCs. B) The quantitative analysis of mature DCs (CD86^+^/CD11c^+^, gated on CD11c^+^ cells) after incubation with different formulations for 24 h. C) TNF-α and D) IL-6 expression level in Raw264.7 cells after incubation with different formulations for 24 h. Data are shown as mean ± standard deviation (SD) (n = 3; *P < 0.05, **P < 0.01, ***P < 0.001).

The antigens could promote the maturation of immature DCs (iDCs) 42, hence, the immune activation was evaluated through assessing the level of maturation of DCs 43. As shown in Figure 5A, firstly, 4T1 cells were cultured in the donor wells (upper wells) with different formulations. Subsequently, iDCs were seeded in the receptor wells (bottom wells) and co-cultured with the upper 4T1 cells for another 24 h. Finally, the DCs were collected to analyze costimulatory molecules CD86 and CD11c, the markers of DCs maturation. In contrast with the free CUR and CaPA group, the ratio of CD86^+^ in CD11c^+^ in CaPCA and VNP group significantly increases (Figure 5B), suggesting both VNP and Ca^2+^ overload could trigger immune activation and induce the maturation of DCs. The group of CaPCAV exhibited the largest ratio of CD86^+^ in CD11c^+^ (Figure 5B) due to the synergistically enhanced immune activation induced by Ca^2+^ overload and VNP.

TNF-α and interleukin 6 (IL-6) are the DC activation-related cytokines, hence the level of TNF-α and IL-6 were also investigated to further assess immune activation 44. Raw264.7 cells treated with CaPCAV generated a larger amount of IL-6 (Figure 5C) and TNF-α (Figure 5D) than other groups, indicating the largest level of DC maturation. All the above results confirmed that Ca^2+^ overload and VNP could significantly improve the cellular immune response.

### *In vivo* bio-distribution

**Figure 6:**
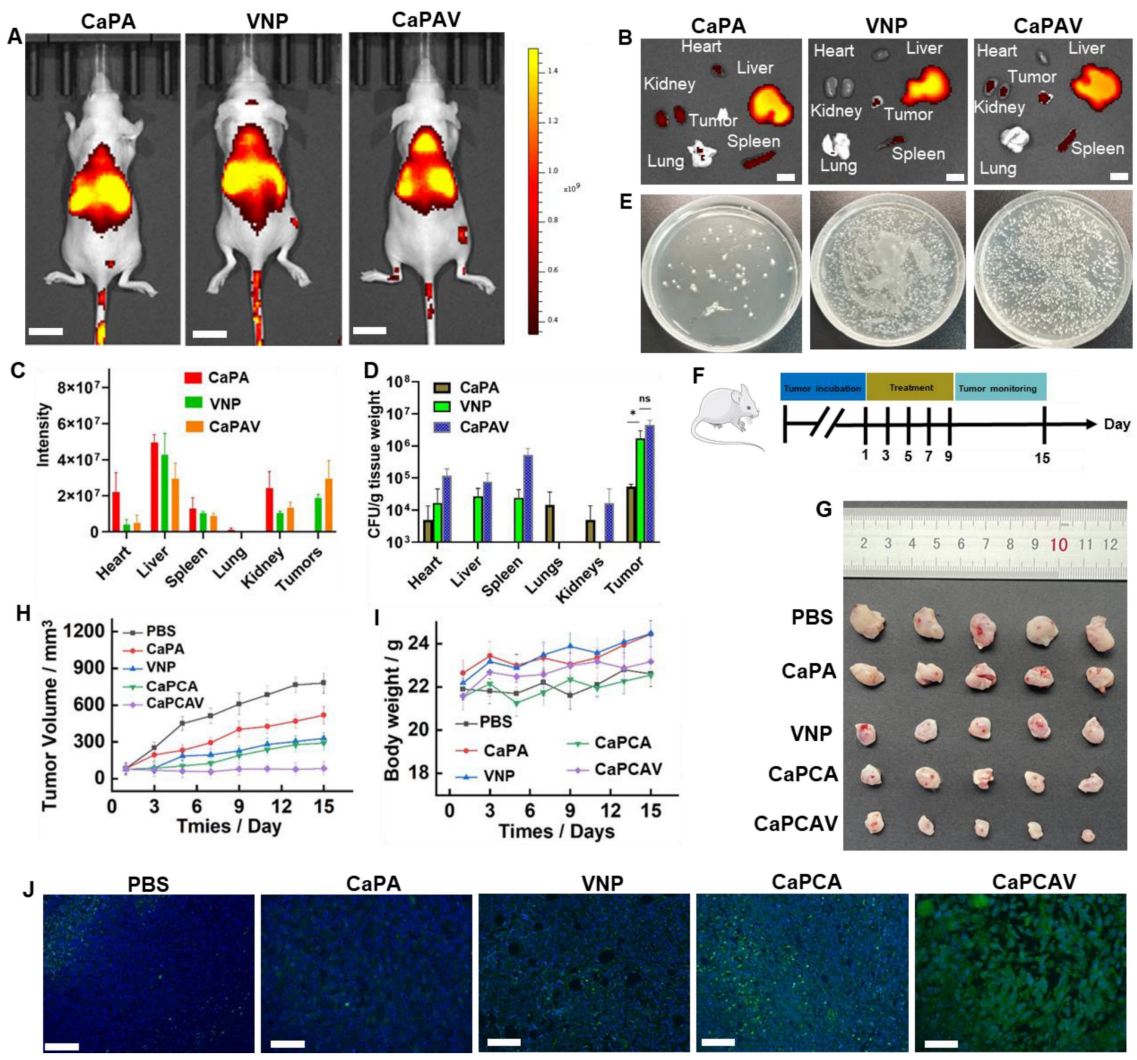
*In vivo* bio-distribution and anti-tumor efficacy. A) Fluorescence imaging of the tumor-bearing mice (the scale bar: 10 mm) and B) ex-vivo imaging and C) the quantitative analysis of harvested organs (the scale bar: 3 mm). D) The amount of VNP in harvested organs. E) The corresponding photographs of VNP in tumor. F) The animal study protocol. G) The tumor images, H) tumor growth profiles and I) body weight changes of 4T1 tumor-bearing mice during the treatment with PBS, CaPA, VNP, CaPCA and CaPCAV, respectively. J) Histological observation of the harvested tumor tissues stained with TUNEL, Scale bar: 50 μm. Data is expressed as the mean ± SD; *P <0.05, **P < 0.01, ***P < 0.001.

The bio-distribution of various formulations was evaluated in 4T1 tumor-bearing mice model (Figure 6A). Cy5.5 exhibit higher intensity of fluorescence than that of CUR, hence, Cy5.5 was selected to replace the CUR. For labeling the VNP, the ADA modified liposome loaded with Cy5.5 was conjugated with VNP-CB^7^ through the CB^7^-ADA host-guest interaction. Among all the organs of CaPA group, liver exhibited the largest fluorescence intensity of Cy5.5 (Figure 6B and 6C), suggesting CaP was mainly metabolized through the liver. In VNP group, the larger intensity of Cy5.5 fluorescence in spleen was observed than other organs, indicating VNP was mainly metabolized through the spleen (Figure 6B). A large fraction of accumulation of VNP in tumors (Figure 6D and 6E) indicated the excellent targeting ability of VNP. Benefiting from the unique property of hypoxia targeting, CaPAV exhibted a higher fluorescence intensity of Cy5.5 than that of CaPA in tumors, confirming the well tumor-targeting efficiency of CaPAV (Figure 6D and 6E).

### *In vivo* anti-tumor therapy

The anti-tumor efficiency was evaluated in 4T1-tumor-bearing mice. The various formulations were injected into the 4T1-tumor-bearing mice on Day 1, 3, 5, 7, 9 via the tail veins, respectively (Figure 6F). The tumors of control group grew more rapidly than other groups (Figure 6G and 6H). VNP exhibited a modest anti-tumor effect, due to bacteria-induced innate and adaptive immune responses 21. Beneficial from the enhanced Ca^2+^ overload induced by CUR, the CaPCA group exhibited better anti-tumor effects than that of CaPA group. CaPCAV group showed the best anti-tumor effects among all the groups, due to the enhanced Ca^2+^ overload and immune response. Moreover, the moderate change of body weight in CaPCAV group suggested the low toxicity of CaPCAV (Figure 5D). The immunofluorescence TUNEL analysis of tumors also confirmed the highest antitumor effects of CaPCAV among all the tested groups (Figure 6J). The above results of anti-tumor efficiency confirmed that CaPCAV could synergistically magnify Ca^2+^ overload and immunotherapy.

### *In vivo* immune system activation

**Figure 7:**
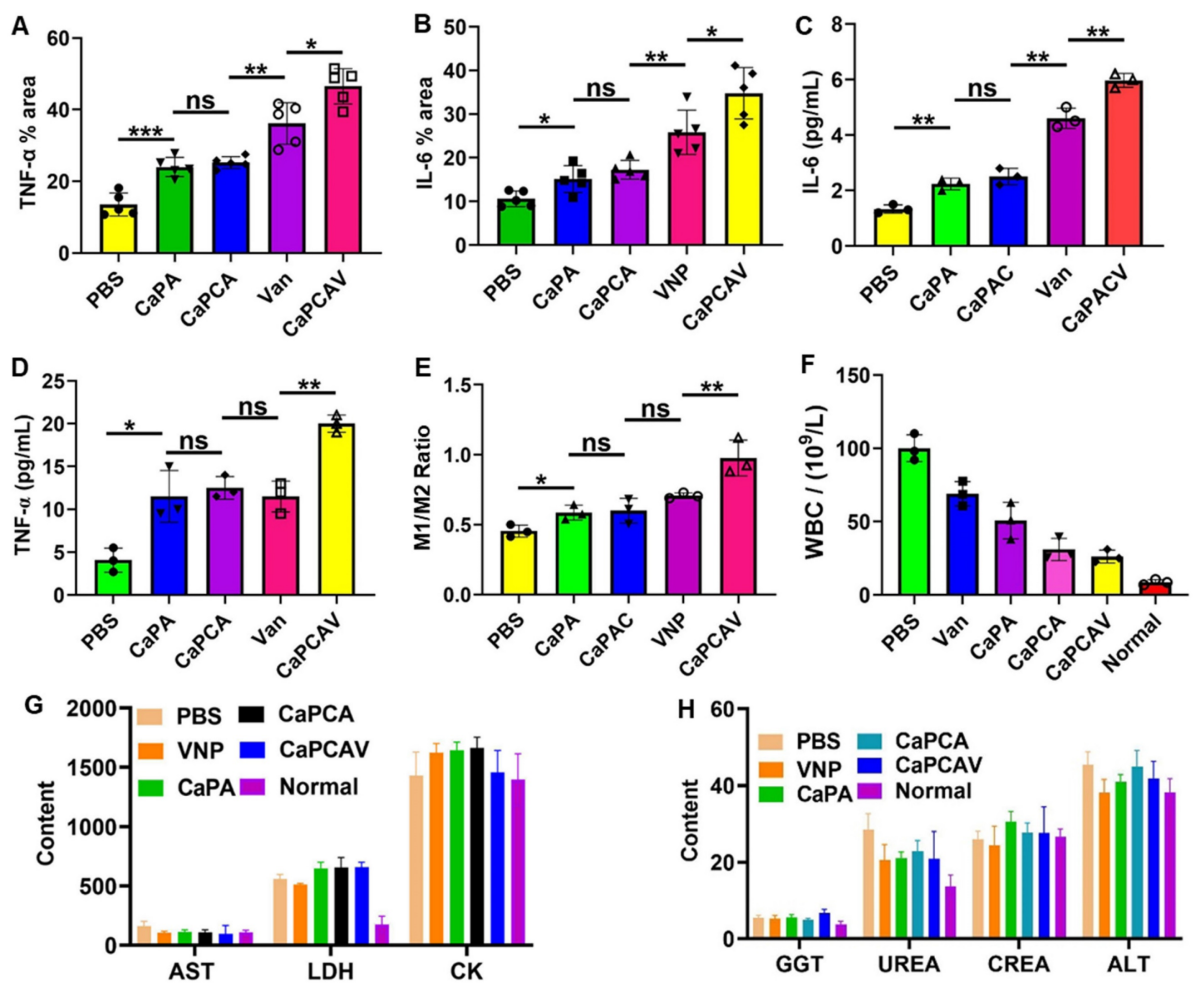
*In vivo* immune response and safety evaluation. A) TNF-α and B) IL-6 quantitative analysis of tumor tissues. The level of C) TNF-α and D) IL-6 in blood samples obtained from the treated mice. E) The ratio of M1/M2 of tumor tissues determined by analysis the ratio of CD11c (an M1 marker) CD206 (an M2 marker). F) WBC, G) AST, LDH and CK, and H) GGT, UREA, CREA and ALT level of blood samples collected from the normal and treated mice. Scale bar: 50 μm. Data is expressed as the mean ± SD (n =3); *P <0.05, **P < 0.01, ***P < 0.001.

The expression level of representative immune cytokines TNF-α and IL-6 was investigated to evaluate the *in vivo* immune response. PBS group exhibited nearly negligible overexpression of TNF-α (Figure 7A) and IL-6 (Figure 7B), while CaPA exhibited high expression of TNF-α and IL-6, due to Ca^2+^ overload induced apoptosis. Attribute to the enhanced Ca^2+^ overload induced by CUR, CaPCA group had higher expression of TNF-α and IL-6 than that of CaPA group. VNP group also upregulated the expression of TNF-α and IL-6 due to the induced innate and adaptive immune responses. The TNF-α (Figure 7C) and IL-6 (Figure 7D) in serum of CaPCAV were also most overexpressed among all the tested groups, which further confirmed that CaPCAV-mediated apoptosis exhibited the highest immune response induced by both Ca^2+^ overload and VNP.

The immune response could induce the polarization of anti-inflammatory macrophages (M2) to pro-inflammatory macrophages (M1), hence, ratio of CD11c (an M1 marker) and CD206 (an M2 marker) was used to further analyze the immune response (Figure 7E). The largest ratio of M1/M2 was exhibited in CaPCAV group, suggesting that tumor cell apoptosis induced by CaPCAV could significantly improve the immune response. DCs could process antigen materials and present them to T cells, resulting in the activation of immune response. Hence, the T cell level in the tumor was also assessed. The immunostaining of CD4^+^ and CD8^+^ were conducted on the tumor sections (Figure S11), an obviously increased intratumor infiltration of T cells was observed in mice treated with CaPCAV, which confirmed the CaPCAV could enhance the infiltration of immune cell into the tumor tissues. The mice treated with formulations containing VNP exhibited an increased number of white blood cells (WBC) than that of normal mice (Figure 7F), due to the mild infections induced by the colonization of VNP.

### *In vivo* safety evaluation

Aspartate aminotransferase (AST), alanine aminotransferase (ALT) and glutamyl transpeptidase (GGT) are the indicators of inflammatory damage of the liver. Lactate dehydrogenase (LDH) and creatine kinase (CK), Urea (UREA) and creatinine (CREA) are the markers of inflammatory damage of the heart and kidneys, respectively. The levels of AST, ALT, GGT, LDH, CK, UREA and CREA in the mice treated with CaPCAV were all within the normal range (Figure 7G and 7H), indicating that the CaPCAV were generally safe and would not induce the damage to the heart, liver and kidneys.

## Conclusions

In conclusion, we have developed a supramolecular conjugate of living bacteria and Ca^2+^ nanogenerators to enhance the targeting efficiency and tumor penetration of nanomedicines for Ca^2+^ overload-related tumor therapy. This approach achieves synergistic Ca^2+^ overload and immunotherapy with improved specificity and effectiveness. Leveraging the excellent tumor-targeting ability of VNP, CaPCAV specifically accumulates at tumor sites and releases CaCO_3_. The combination of CaCO_3_ and CUR induces significant Ca^2+^ overload in tumor cells, leading to mitochondrial destruction and inhibition of tumor growth. Moreover, VNP colonization promotes immune responses, enhancing the efficiency of immunotherapy. The developed supramolecular conjugate of CaCO_3_ and VNP mutually amplifies Ca^2+^ overload and immunotherapy, thereby improving the therapeutic efficiency of tumors. This work not only introduces a novel method for utilizing supramolecularly engineered bacteria as stable, targeted drug carriers but also provides insights into tumor therapy with enhanced specificity and efficacy.

### Statistical analysis

Statistical analysis relied on one-way and two-way ANOVA methods. Statistical significance was annotated with *P ≤ 0.05, **P ≤ 0.01 and ***P ≤ 0.001, respectively. All data were presented as the mean value ± standard deviation of independent runs.

## Supplementary Material

Supplementary materials and methods, figures.

## Figures and Tables

**Figure 1:**
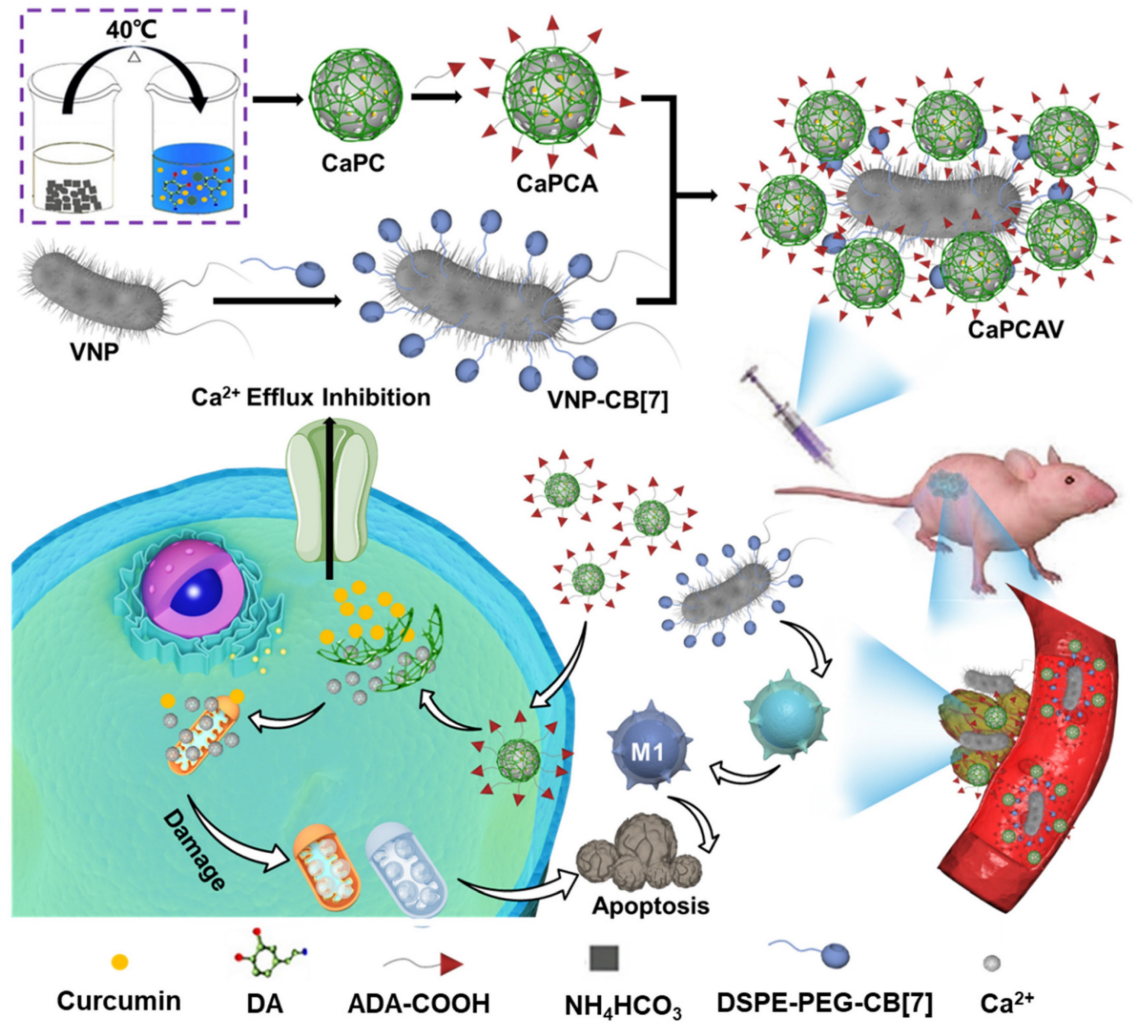
The construction and anti-tumor CaPCAV. A) Scheme of the preparation of CaPCAV. B) The specific delivery of CaPCAV and subsequent Ca^2+^ overload and immunotherapy against tumors.
