# Supplementary material for: Supramolecularly engineered bacteria mediated calcium overload and immunotherapy of tumors
Source: Theranostics. 2024 Oct 7;14(17):6560–70. doi: 10.7150/thno.99931 (PMC11519789; doi:10.7150/thno.99931)
Supplement: Supplementary file 1 — Supplementary materials and methods, figures. [file thnov14p6560s1.pdf]

Supramolecularly Engineered Bacteria Mediated Calcium-Overload and Immunotherapy of Tumors

Beibei Xie, Linmiao Dong, Leo Wang, Ruibing Wang and Chunlai Li

Materials

Calcium chloride dihydrate ($CaCl_2 \cdot 2H_2O$), ammonium bicarbonate (NH_4HCO_3), dopamine (DA), dicyclohexylcarbodiimide (DCC) and 1-ethyl-3-(3-dimethylaminopropyl)-carbodiimide (EDC) were purchased from Sigma-Aldrich Co. DMEM (Dulbecco's Modified Eagle Medium) medium, Fetal Bovine Serum (FBS), Trypsin-EDTA, Trypsin, and McCoy's 5A Medium were purchased from HyClone/Thermo fisher (Beijing, China). DSPE-PEG(2000)-SH, ADA modified with carboxyl (COOH-ADA) were bought from Xi'an Ruixi Biological Technology Co., Ltd. RPMI 1640 medium, Fetal Bovine Serum (FBS), Trypsin-EDTA, and Trypsin were purchased from HyClone/Thermo fisher (Beijing, China). Fluo-4 AM, calcium ionophore (Ionomycin), 2-(4-amidinophenyl)-6-indolecarbamidine dihydrochloride (DAPI), Hoechst 33342, Ca^{2+} assay kit, adenosine triphosphate (ATP) assay kit, mitochondrial membrane potential assay kit with JC-1, Alexa Fluor 647-labeled goat anti-Rabbit IgG (H+L), Alexa Fluor 647-labeled goat anti-mouse IgG (H+L), cytochrome C rabbit and mouse monoclonal antibody were purchased from Beyotime Biotechnology Inc. (Shanghai, P. R. China). Anti-mouse CD11c antibody, anti-mouse CD206 antibody, anti-mouse CD80 antibody, and anti-mouse CD86 antibody were gotten from BioLegend, Inc. Lipopolysaccharide (LPS) was obtained from Sigma Aldrich (USA). CB[7] was synthesized in our laboratory. All the chemicals were analytic grades and used without further purification. Attenuated VNP was bought from the American Type Culture Collection.

Methods

Synthesis of DSPE-PEG-CB[7]

DSPE-PEG-CB[7] was synthesized refer to our previously reported method through the click reaction between monoallyloxy CB[7] and DSPE-PEG-SH[1]. Briefly, DSPE-PEG-SH (1 mM) was mixed with monoallyloxy CB[7] (1 mM) and dissolved in deionized water, subsequently irradiated by UV light at room temperature for 24 h. After the dialysis purification (M_W = 2000 Da), the DSPE-PEG-CB[7] was obtained.

Preparation of VNP-CB[7]

VNP was planted on the LB agar plate and inoculated overnight at 37°C. Afterward, the VNP colony was picked, diluted into a 5 mL culture medium, and grown overnight to reach an OD600 of about 0.4. Then VNP was washed with PBS by centrifugation (2300 *g*, 3 min) and re-suspended in PBS with 100 μM of DSPE-PEG-CB[7] for 6 h. The VNP-CB[7] was obtained after centrifugation and washing with PBS, and the pellet was suspended with PBS at OD600 of about 0.4 for further use.

Preparation of CaPC nanoparticles

CaPC was synthesized by a reported one-pot gas diffusion process[2]. Briefly 150 mg of $CaCl_2 \cdot 2H_2O$, 2 mg of dopamine and 20.0 mg of CUR were dissolved in a beaker filled with 100 mL of absolute ethanol; then the mixture was placed in an air tight container with 5 g of NH_4HCO_3 and heated at 40 °C for 24 h. At last, CaPC was collected and purified by centrifugation (5000 *g*) and washed with ethanol and water, respectively.

The preparation of CaPCA

The ADA-PDA conjugate was synthesized via esterification between ADA-COOH and PDA according to the proposed synthetic route (Figure S2). Briefly, 10 mg of CaPCA nanoparticles were dispersed in anhydrous DMSO with 16.5 mg of EDC and 12.4 mg of DCC, stirring for 30 min at room temperature (RT). 25 mg of FA were added to the above solution for reacting 48 h. The CaPCA was collected and purified by centrifuged (5000 *g*) and washed with DMSO, ethanol and water, respectively.

Preparation of CaPCAV

1mL CaPCAV (100 $\mu g \cdot mL^{-1}$) was mixed with VNP-CB[7] (10^6 CFU) in PBS buffer and incubated at 37 °C for 30 min. The CaPCAV was gained through the host-guest interaction between ADA and CB[7]. Later, the mixture was washed three times by PBS through centrifugation, and re-suspended in PBS buffer for further use.

Nanoparticle characterization

SU-8010 (Hitachi, Japan) under the operating voltage of 3 kV was applied for SEM characterization. JEM-1011 TEM (JEOL, Japan) with an accelerating voltage of 100 kV was used for TEM imaging. The TEM species were prepared by dropping 5 μL of suspensions (0.05 $mg \cdot mL^{-1}$) on the copper grid and then air drying. The hydrodynamic diameters and zeta potential of samples were analyzed on the DLS (Malvern Zetasizer Nano ZS). The iron content was determined on the Thermo iCAP Qs ICP-MS.

Drug release behavior

The amount of CUR was measure by UV-Vis absorption spectra of CUR. The loading ratio of CUR in CaPC was calculated from the formula: Loading ratio = (the amount of CUR inside the CaPC) / (the total amount of the CaPC) ×100%. To analyze the drug release behavior of CUR, 2 mg of CaPCA and CaPCAV were suspended in 2 mL of PBS buffer solution at different pH of 7.4, 6.5, and 5.5, respectively, to yield 1 mg/mL suspension, and the suspensions were transferred into dialysis bags (M_D=1000 Da). The dialysis bags were soaked in 20 mL of release medium (buffer with different pH values), respectively, and stirred gently at room temperature. At different time points, including 0, 0.25, 0.5, 1, 2, 3, 4, 6, 8, 10, 12, and 24 h, 1 mL of release solution was collected for absorption measurement, and 1 mL fresh buffer solution at different pH values was supplemented to keep release medium volume constant. UV-Vis absorption spectra of CUR was used to calculate the SAS concentration. The release amount of SAS in the dialysis bag at each time point was measured by DR600 UV-Vis

Spectrophotometer. Each experiment was performed in triplicate.

In vitro biocompatibility evaluation

MTT assay was used to assess the biocompatibility of nanoparticles against LO2 cells. First, LO2 cells were inoculated in a 96-well plate with a density of 10^4 cells/well and cultured overnight. Then, CaPA, CaPCA, and CaPCVA with different concentrations of CaP (0, 12.5, 25, 50, and 100 $\mu g \cdot mL^{-1}$) were added and treated for 24 h. Later, the culture medium was discarded, and 100 μL culture medium involving MTT (5 $mg \cdot mL^{-1}$) was applied to each well, subsequently incubated for another 4 h. Then the medium was discarded, followed by adding 100 μL DMSO to dissolve formazan. Eventually, the cytotoxicity of nanoparticles to LO2 cells was assessed through a Microplate reader.

In vitro cytotoxicity assay

Similarly, an MTT assay was applied to investigate the cytotoxicity of different formulations against 4T1 cells. First, 4T1 cells were inoculated in a 96-well plate with a density of 10^4 cells/well and incubated overnight. When cells reached around 70% confluence, cells were incubated with PBS, VNP, CUR, CaPA, CaPCA and CaPCAV containing the same concentration of CUR (20 $\mu g \cdot mL^{-1}$), CaP (100 $\mu g \cdot mL^{-1}$) and VNP (10^6 CFU). After treated for 24 h, the culture medium was removed, and the FBS-free medium involving MTT (5 $mg \cdot mL^{-1}$) was added and incubated for another 4 h. Then the medium was discarded, followed by adding 100 μL DMSO to each well to dissolve formazan. Eventually, the cytotoxicity of different groups against 4T1 cells was assessed by a Microplate reader at 490 nm.

Investigation of the cell apoptosis induced by the Ca^{2+} overload

4T1 cells were first seeded in the confocal petri dishes (35 mm) at a density of 1.0 × 10^5 cells/well and cultured in 1.0 mL total DMEM medium overnight. Then CaPA, CaPCA and CaPCAV containing the equivalent concentration of CaP (100 $\mu g \cdot mL^{-1}$) were added to the confocal dishes. After 12 h incubation, the cells were stained with a Ca^{2+} probe (Fluo-4 AM) and Hoechst 33342 and washed with PBS. Finally, cells were immobilized in 4% paraformaldehyde and imaged by CLSM. 4T1 cells were first seeded at a density of 1.0 × 10^4 cells/well and cultured in 100 μL total DMEM medium overnight. After the CaPCAV (50 $\mu g \cdot mL^{-1}$) was added, the 100 μL PBS (1 mM), $CaCl_2$ (1 mM), Ionomycin (5 μM), BAPTA-AM (50 μM) was added, respectively and incubated for 12 h. After discarding the medium, 100 μL fresh medium containing 10 μL MTT solution (5 $mg \cdot mL^{-1}$) was added to each well, and cultured for 4 h. And then, 100 μL DMSO was added to dissolve formazan after the supernatant was discarded. Eventually, the cytotoxicity of nanoparticles to 4T1 cells was assessed by a Microplate reader.

Detection of Mitochondrial dysfunction

4T1 cells were first seeded in the confocal petri dishes (35 mm) at a density of 1.0 ×

10^5 cells/well and cultured in 1.0 mL total DMEM medium overnight. Then the CaPA, CaPCA and CaPCAV containing the equivalent concentration of CaP (100 μg·mL^{-1}) were added to the confocal dishes. After incubation for 12 h, the cells were stained with the mitochondrial probes (Mito Tracker® Red CMXRos) or mitochondrial membrane potential probes (JC-1) and Hoechst 33342 and washed with PBS. Finally, the cells were fixed in 4% paraformaldehyde and CaDtured by CLSM.

Characterization of the different types of $CaCO_3$ in cells

The 4T1 cells were first inoculated in 2.0 mL full DMEM medium at a density of 5.0 × 10^5 cells/well overnight, and then the CaPA, CaPCA and CaPCAV containing the equivalent concentration of CaP (100 μg·mL^{-1}) were added to each well. After 12 h culture, cells and medium were harvested and centrifuged (500 *g*, 3min) to obtain the cell precipitates. On the one hand, the cell sediments were then dehydrated, embedded, and sliced to observe the morphology of cells and nanoparticles by a bio-TEM.

***In vitro* DCs maturation experiment**

Bone marrow-derived iDCs were harvested from BALB/c mice aged 6 to 8 weeks. The 24-well transwell system with a 0.4 μm polycarbonate porous membrane was used to study *in vitro* DCs maturation experiment. First, 2×10^4 4T1 cells per well were inoculated in the upper well. Subsequently, cells were incubated with PBS, CUR, CaPA, VNP, CaPCA and CaPCAV containing the equivalent concentration of CaP (100 μg·mL^{-1}) with an equivalent concentration of CUR (20 μg/mL), CaP (100 μg/mL), or VNP (10^6 CFU) for 24 h. After that, the upper well was transferred and co-incubated with DCs previously inoculated in the bottom well. After co-culture for 24 h, DCs in the bottom were harvested and stained with anti-CD11c antibody and anti-CD86 antibody for 1 h. Later, DCs were resuspended by PBS and assessed by flow cytometry after washing by centrifugation.

***In vitro* immune analysis**

The TNF-α and IL-6 levels were evaluated using ELISA kits (Lai Er Bio-Tech, China) in a 24-well transwell system with a 0.4 μm polycarbonate porous membrane. First, 4T1 cells were inoculated in the upper well. When the cells reached 70-80% confluence, PBS, CUR, CaPA, VNP, CaPCA and CaPCAV containing the equivalent concentration of CaP (100 μg·mL^{-1}) with an equivalent concentration of CUR (20 μg·mL^{-1}), CaP (100 μg·mL^{-1}), or VNP (10^6 CFU) for 24 h. Afterward, the upper wells were transferred and co-incubated with Raw 264.7 previously inoculated in the bottom well. After the co-culture, the supernatant was obtianed for cytokine TNF-α and IL-6 analysis by ELISA assay kits.

***In vivo* bio-distribution study**

Female BALB/c mice (6 to 8 weeks) were obtained from the Animal Center of the University of Macau. 10^6 4T1 cells were subcutaneously injected to the hind legs of mice and observed for 12 days. Then, the mice were separated into 5 groups at random (*n*=3), and the free Cy5.5, CaPA, VNP, CaPAV were intravenously injected into the mice through the tail vein (2 mg/kg Cy5.5). At predetermined time points after injection (0, 6, 12, 24, 36, and 48 h), mice were anesthetized, and an *in vivo*

imaging system (IVIS) was applied to capture fluorescence signals. All mice were sacrificed 48 hours later to collect the organs and tumor for fluorescence imaging.

Later, 0.02 mg tissue was cut from the organs or tumors for grinding to observe the distribution of Ca in different organs.

Similarly, 0.02 mg tissue was cut from the organs or tumors for grinding to observe the distribution of VNP in different organs. Single-cell suspension was obtained using a Falcon® 40 μm cell strainer. After centrifuging (300 *g*, 5 min), the supernatant was diluted at different times for plating and colony counting. Later, 100 μL bacteria solution was added to the agar plate, spread uniformly, and incubated at 37℃ overnight for colony counting.

In vivo **anti-tumor study**

Female BALB/c mice (6 to 8 weeks) were obtained from the Animal Center of the University of Macau. 10^6 4T1 cells were injected to the hind legs to get 4T1 tumor-bearing mice. Once the tumor volume reached about 80 mm^3, the mice were separated into 5 groups at random and intravenously injected with 0.1 mL PBS, VNP, CaPA, CaPCA and CaPCAV with an equivalent CaPA of (100 $mg \cdot mL^{-1}$) on day 1, 3, 5, 7, 9. The loading amount of CaPA nanoparticles was about 1.5 mg per 10^5 CFU of VNP, the dose of VNP is about 6.7×10^5 CFU, which is consistent the used dose in reported literatures.[3,4] The tumor volume and mice weight were recorded every 3 days until the test ended. The tumor volume was calculated as the following formula: $V = (L \times W^2)/2$, where L and W denoted the maximum and minimum diameters. When the 14-day treatment ended, the mice were sacrificed to harvest their major organs and tumors for H&E and TUNEL staining, Ca and VNP distribution evaluation, the study of M1/M2 macrophage ratio in tumor section, and IL-6 and TNF-α expression level in the tumor site. The blood was obtained from the heart and eyeball of sacrificed mice for further analysis.

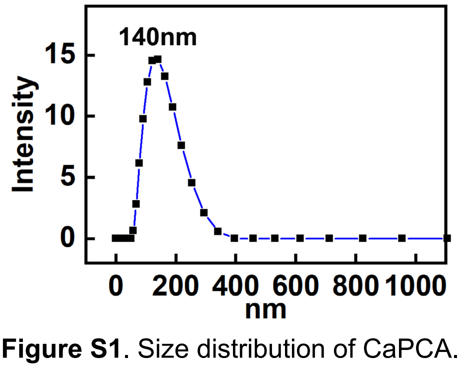

Figure S1. Size distribution of CaPCA.

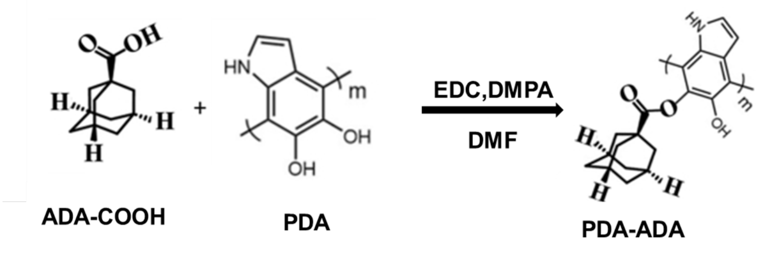

Figure S2. Synthetic route of CaPCA.

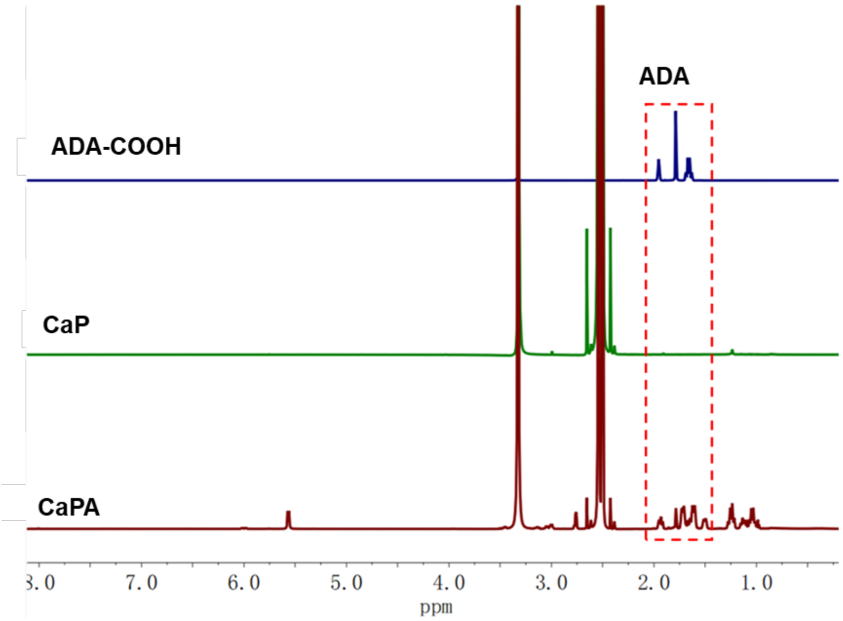

Figure S3. ^{1}H NMR spectrum of ADA-COOH, CaP and CaPA.

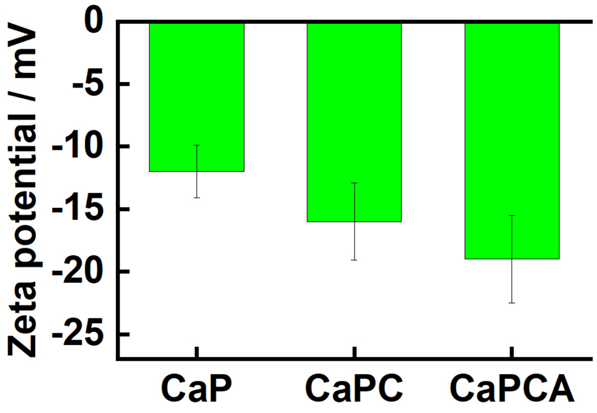

Figure S4. The Zeta potential of CaP, CaPC, and CaPCA.

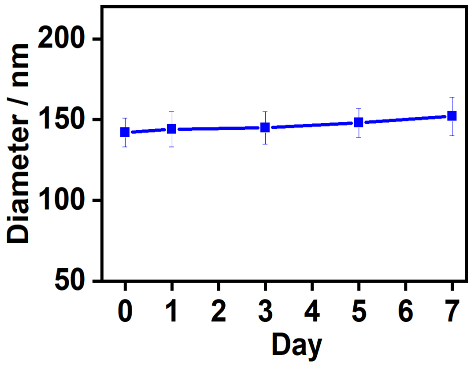

Figure S5. The mean hydrodynamic diameters of CaPCA after incubation in PBS for different durations determined by DLS.

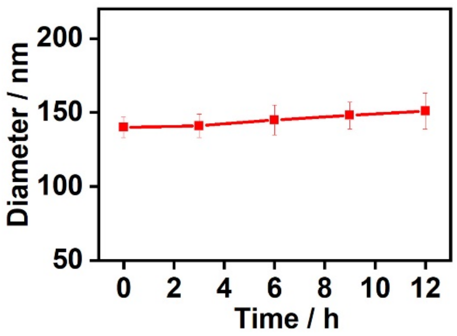

Figure S6. The mean hydrodynamic diameters of CaPCA after incubation in 10% FBS for different durations determined by DLS.

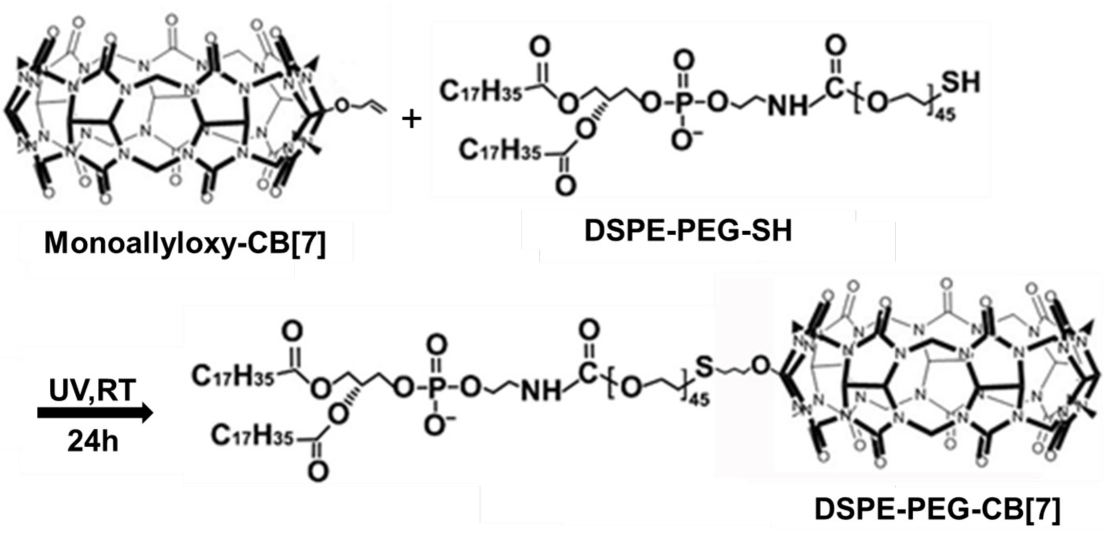

Figure S7. Synthetic route of DSPE-PEG-CB[7].

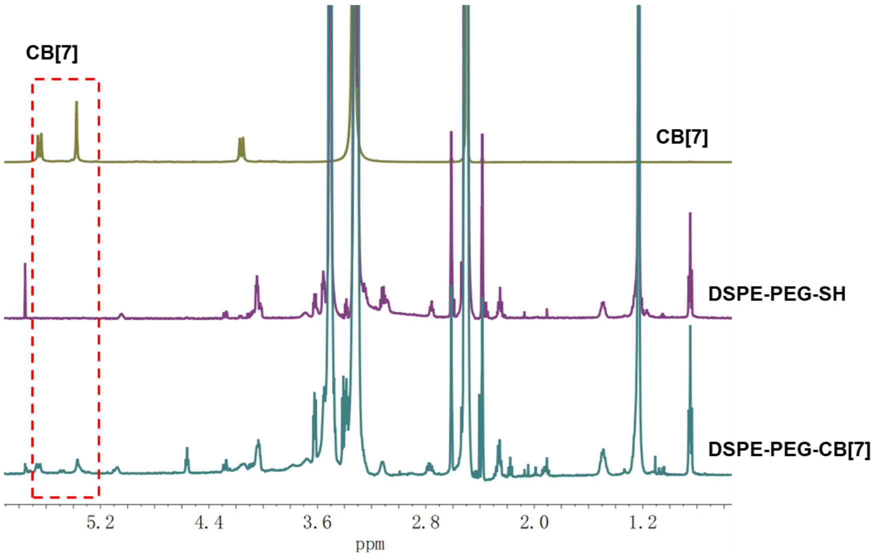

Figure S8. ^{1}H NMR spectrum of CB[7], DSPE-PEG-SH, and DSPE-PEG-CB[7].

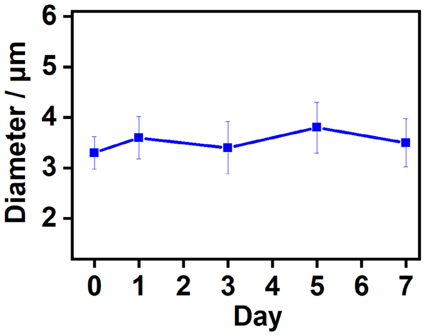

Figure S9. The mean hydrodynamic diameters of CaPCAV after incubation in PBS for different durations determined by DLS.

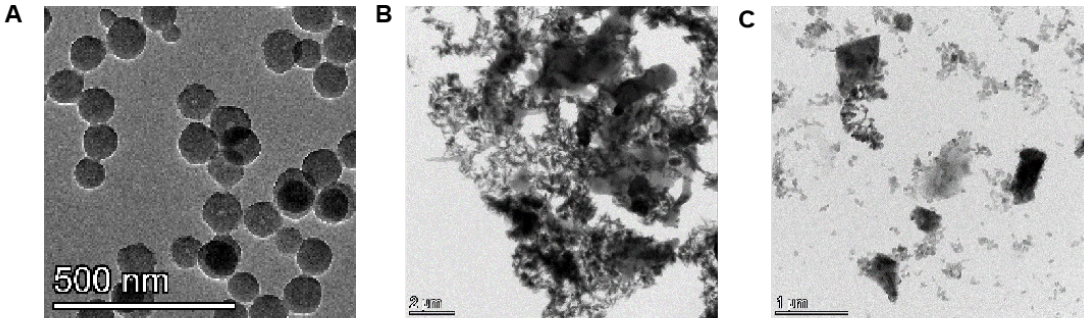

Figure S10. TEM images of CaPCA after incubation in PBS at different pH 5.5, 6.5, and 7.4 for 24 h.

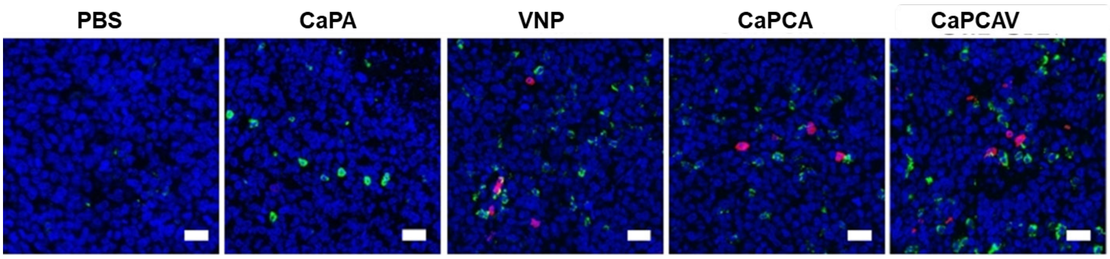

Figure S11. Immunostaining of the CD4 (green) and CD8 (red) tumor tissues collected from mice treated with PBS, CaPA, VNP, CaPCA and CaPCVA. Scale bar: 20 μm.

References

1. Gao C, Cheng Q, Li JY, Chen J, Wang QF, Wei JW, et al. Supramolecular Macrophage-Liposome Marriage for Cell-Hitchhiking Delivery and Immunotherapy of Acute Pneumonia and Melanoma, Adv Funct Mater. 2021; 31: 2102440-53.
2. Zhao H, Zhu WW, Liu JJ, Liang C, Zhang Q, Liu Z. Synthesis of hollow biomineralized $CaCO_3$–polydopamine nanoparticles for multimodal imaging-guided cancer photodynamic therapy with reduced skin photosensitivity, J Am Chem Soci. 2018; 140: 2165-78.
3. Chen WF, Wang Y, Qin M, Zhang XD, Zhang ZR, Sun X, Bacteria-driven hypoxia targeting for combined biotherapy and photothermal therapy, ACS Nano. 2018; 12:

5995-6005.
4. Yi X, Zhou H, Chao Y, Xiong S, Zhong J, Chai Z, et al. Bacteria-triggered tumor-specific thrombosis to enable potent photothermal immunotherapy of cancer. Sci Adv. 2020; 6: eaba3546-57.
